# Circulating factors induced by time-restricted eating drive metabolic reprogramming in endothelial cells

**DOI:** 10.1007/s11357-026-02425-2

**Published:** 2026-07-24

**Authors:** Shoba Ekambaram, Tamas Kiss, Zoltan Ungvari, Kiana Vali Kordestan, Dorina Nagy, Rebeka Kristof, Raghavendra Yelahanka Nagaraja, Tripti Gautam, Roland Patai, Rafal Gulej, Siva Sai Chandragiri, Anna Ungvari, Balázs Győrffy, Zoltan Benyo, Andriy Yabluchanskiy, Stefano Tarantini, Anna Csiszar

**Affiliations:** 1 Vascular Cognitive Impairment, Healthy Brain Aging Program, Department of Neurosurgery, University of Oklahoma Health Sciences Center, NeurodegenerationOklahoma City, OK USA; 2 Pediatric Center, Semmelweis University, Budapest, Hungary; 3 Neurocognitive Diseases Research Group, HUN-REN-SU Cerebrovascular, 1094 Budapest, Hungary; 4 Institute for Translational Research, Budapest, Hungary; 5 International Training Program in Geroscience, Doctoral College, Health Sciences Division/Institute of Preventive Medicine and Public Health, Semmelweis University, Budapest, Hungary; 6 Oklahoma Center for Geroscience and Healthy Brain Aging, University of Oklahoma Health Sciences Center, Oklahoma City, OK USA; 7 Institute of Preventive Medicine and Public Health, Semmelweis University, Budapest, Hungary; 8 Fodor Center for Prevention and Healthy Aging, Semmelweis University, Budapest, Hungary; 9 Department of Bioinformatics, Pediatric Center, Semmelweis University, Budapest, Hungary; 10 Institute of Transdisciplinary Discoveries, Medical School, University of Pecs, Pecs, Hungary; 11 Cancer Biomarker Research Group, Institute of Molecular Life Sciences, Hungarian Research Network, Budapest, Hungary; 12 Institute of Clinical Pathophysiology, Semmelweis University, Budapest, Hungary; 13 Stephenson Cancer Center, University of Oklahoma, Oklahoma City, OK USA

**Keywords:** Time-restricted eating, Endothelial cell, Vascular cognitive impairment and dementia, VCID, Microcirculation, MTORC1, Mitochondrial function, GDF15, Endothelial dysfunction, Vascular aging, Transcriptomics, Nutrient sensing, Cognitive decline, Aging, Brain, GSEA

## Abstract

Age-related endothelial dysfunction in the cerebral microcirculation contributes significantly to the pathogenesis of vascular cognitive impairment and dementia (VCID). Time-restricted eating (TRE) has emerged as a promising lifestyle intervention with beneficial effects on metabolic and vascular health; however, the mechanisms by which TRE influences the brain microvasculature remain incompletely understood. In particular, the role of circulating factors induced by TRE in modulating endothelial function has not been systematically investigated. Here, we tested the hypothesis that circulating factors derived from humans practicing time-restricted eating (TRE) induce protective and adaptive responses in human cerebromicrovascular endothelial cells. Using a serum transfer bioassay, endothelial cells were treated with serum obtained from aged individuals with or without TRE, followed by transcriptomic profiling. We demonstrate that TRE-associated serum elicits a robust and coordinated transcriptional reprogramming in human cerebromicrovascular endothelial cells, characterized by activation of stress-responsive and metabolic pathways and suppression of anabolic programs. Gene set enrichment analysis revealed significant activation of the integrated stress response (ISR)/ATF4 axis and suppression of mTORC1 signaling, consistent with a shift toward a catabolic, stress-adaptive state. These changes were accompanied by marked induction of the stress-responsive cytokine *GDF15*. At the mitochondrial level, TRE serum promoted increased expression of mitochondrial DNA-encoded oxidative phosphorylation components without activation of canonical mitochondrial biogenesis pathways, suggesting functional remodeling. Upstream regulator analysis identified coordinated activation of stress- and metabolism-associated transcription factors, including ATF4, FOXO, and KLF family members, alongside inhibition of anabolic regulators such as SREBF1/2. Notably, canonical endothelial functional programs, including autophagy and blood–brain barrier maintenance, were not coordinately activated. While individual angiogenesis-related genes were modestly upregulated, these changes did not translate into a coordinated pathway-level response. Collectively, these findings demonstrate that circulating factors induced by TRE promote a distinct endothelial phenotype characterized by metabolic reprogramming and stress adaptation rather than classical inflammatory or reparative responses. This work provides new mechanistic insight into how lifestyle interventions may influence cerebrovascular aging and identifies circulating factors as key mediators linking systemic metabolic state to endothelial function.

## Introduction

Vascular cognitive impairment and dementia (VCID) represent a major and growing public health challenge, accounting for a substantial proportion of age-related cognitive decline [1–6] and frequently coexisting with neurodegenerative pathologies [1]. Central to the pathogenesis of VCID is dysfunction of the cerebral microcirculation, which plays a critical role in maintaining adequate cerebral blood flow and blood–brain barrier (BBB) integrity [7–10]. Age-related impairments in cerebromicrovascular function, including endothelial dysfunction, capillary rarefaction and BBB disruption, are increasingly recognized as key drivers of cognitive decline [7–10]. Interventions that preserve or restore microvascular function by targeting fundamental aging processes therefore represent a promising strategy to mitigate VCID [6, 11–13]. Among these, time-restricted eating (TRE), a dietary pattern that confines food intake to a defined daily time window, has emerged as a potential modulator of aging-related processes [11, 14, 15]. Preclinical studies have shown that TRE improves metabolic homeostasis, enhances vascular function, and promotes both organismal and cellular stress resilience [14, 16, 17]. Emerging evidence further suggests that TRE may positively influence the cerebral microcirculation, improving endothelial function and neurovascular coupling, and may confer benefits for cognitive performance in aging models [11, 15]. However, the mechanisms by which TRE exerts these effects on the brain microvasculature remain incompletely understood.

A growing body of work in geroscience indicates that circulating factors are key mediators of systemic aging phenotypes and responses to metabolic interventions [18–23]. Dietary interventions, including caloric restriction (CR) and TRE, induce profound changes in the circulating milieu, encompassing metabolites, hormones, cytokines, and other signaling molecules [24–30]. These circulating factors can act on peripheral tissues to coordinate organism-wide adaptations [19]. Previous studies have demonstrated that serum derived from animals subjected to CR confers multifaceted anti-aging effects when applied to cultured cells [26, 27, 30, 31]. In particular, work by de Cabo and others has shown that serum from CR rodents and non-human primates (rhesus macaques) enhances endothelial stress resistance, improves endothelial function, and promotes a more resilient cellular phenotype [26, 27, 30, 31]. These findings support the concept that circulating factors can transmit systemic longevity signals and confer multifaceted endothelial protective effects [19].

While TRE does not necessarily reduce caloric intake, it engages overlapping nutrient-sensing and stress-response pathways with CR, including modulation of mTOR signaling, activation of stress-adaptive pathways, and alterations in metabolic flexibility [32–37]. This raises the possibility that TRE-induced changes in circulating factors may similarly influence endothelial function. However, whether circulating factors derived from humans practicing TRE exert protective or adaptive effects on the cerebral microvasculature has not been directly investigated.

This question is particularly relevant in the context of VCID, where endothelial dysfunction and impaired adaptive responses are central to the pathogenesis of the disease [2, 5]. Circulating signals induced by metabolic interventions may represent a critical interface through which systemic metabolic states influence brain aging. This framework suggests that lifestyle interventions such as TRE could modulate cerebrovascular health through blood-borne factors that reprogram endothelial function.

In the present study, we tested the hypothesis that circulating factors derived from humans practicing TRE) induce protective and adaptive responses in human cerebromicrovascular endothelial cells. Using a serum transfer approach [26, 31], endothelial cells were treated with serum obtained from aged individuals with or without TRE, followed by transcriptomic profiling. We demonstrate that TRE-associated circulating factors elicit a robust and coordinated endothelial response characterized by activation of stress-adaptive and metabolic pathways, suppression of anabolic processes, and remodeling of mitochondrial function. These findings provide new insight into the systemic mechanisms by which TRE may influence brain microvascular function and highlight circulating factors as important mediators linking metabolic state to endothelial function and cerebrovascular health.

## Methods

### Human cerebral microvascular endothelial cell culture and serum treatment

Human cerebral microvascular endothelial cells (hCMEC/D3; Sigma-Aldrich, St. Louis, MO, USA; Cat# SCC066) were used as an in vitro model of human cerebral microvascular endothelium. Cells were cultured at 37 °C in a humidified atmosphere with 5% CO₂. Culture vessels were pre-coated with attachment factor solution (Cell Systems, Kirkland, WA, USA; Cat# 4Z0-210) for 15–20 min at 37 °C to promote cell adherence.

Cells were maintained in Complete Classic Medium with serum (Cell Systems), supplemented with CultureBoost™, penicillin–streptomycin (10,000 U/mL; Gibco), and Bac-Off antibiotic (1 mL per 500 mL; Cell Systems), and used at approximately passage 5. For experiments, cells were seeded at a density of 4 × 10 [5] cells per well in six-well plates and allowed to adhere for 24 h.

At ~ 50–60% confluency, cells were treated with 10% human serum diluted in Endothelial Basal Medium (Cell Applications, San Diego, CA, USA; Cat# 100–500) for 48 h [26, 31]. Serum samples were obtained from male and female participants across two groups: older control individuals and older individuals practicing time-restricted eating (TRE). Participants in the control and TRE groups were aged 58–81 years. Individuals in the TRE group adhered to a daily time-restricted eating regimen, with food intake confined to an approximately 6 to 10-h window for a minimum of 26 weeks prior to sample collection. Prior to use, serum samples were heat-inactivated (56 °C for 30 min) to inactivate complement [26, 31]. Following treatment, cells were harvested for downstream analyses, including RNA isolation and transcriptomic profiling.

### Viable cell sorting and RNA isolation

After 48 h of serum treatment, cells were harvested by trypsinization and pelleted by centrifugation (700 × g, 10 min, 4 °C). Cell pellets were resuspended in Endothelial Basal Medium and stained with SYTOX™ Red nucleic acid stain (Invitrogen) to discriminate non-viable cells.

Viable cells were isolated using the WOLF Cell Sorter (NanoCellect) under low-pressure conditions (500 events/µL), enabling removal of debris and dead cells. Sorted cells were subsequently centrifuged (1000 × g, 10 min, 4 °C), pelleted, and stored at –80 °C until processing.

Total RNA was extracted using the QIAcube Connect MDx system (QIAGEN, Germany) following the manufacturer’s protocol. Briefly, frozen cell pellets were thawed, mechanically disrupted, and lysed in RLT buffer prior to automated spin column-based purification.

RNA quantity and purity were assessed using a NanoDrop spectrophotometer, and integrity was evaluated using an Agilent TapeStation system. RNA integrity was uniformly high across all samples (RIN range: 9.8–10.0), indicating excellent RNA quality suitable for transcriptomic analysis.

### Library preparation and RNA sequencing

cDNA libraries were prepared from high-quality total RNA using a stranded mRNA library preparation protocol (poly(A) enrichment), according to the manufacturer’s instructions (Illumina-compatible workflow). Briefly, polyadenylated transcripts were enriched from total RNA, fragmented, and reverse-transcribed to generate first-strand cDNA, followed by second-strand synthesis. Double-stranded cDNA fragments were end-repaired, A-tailed, and ligated to indexed sequencing adapters. Libraries were amplified by PCR and purified prior to quality assessment. Library size distribution and concentration were evaluated using an Agilent TapeStation system. Indexed libraries were pooled at equimolar concentrations and sequenced at the OUHSC Genomics Core Facility on an Illumina NextSeq 2000 platform using sequencing-by-synthesis chemistry to generate 151-bp paired-end reads. Sequencing was performed to a target depth of approximately 30 million reads per sample, yielding an average depth of 30.8 million reads per sample (range: 15.4–39.4 million reads).

### RNA-Seq data processing and quantification

Raw sequencing reads were assessed for quality using FastQC (v0.12.1) and MultiQC (v1.31). Transcript abundance was quantified via pseudo-alignment to the human reference transcriptome (GRCh38) using Kallisto (v0.48.0) [38]. All downstream analyses were conducted in the R environment (v4.5.2).

### Quality control and pre-processing

Transcript-level estimates were imported and summarized to gene-level counts using tximport (v1.38.2) [39]. Lowly expressed genes were filtered, retaining those with at least 10 counts in a minimum of three samples. Sample-level quality control was performed using Principal component analysis (PCA) and sample-to-sample Pearson correlation analysis.

Based on combined assessment of Principal component analysis and sample-to-sample correlation matrices, five samples demonstrating marked separation from their biological group and reduced correlation with other samples were identified as outliers and excluded from downstream analyses. Exclusion was not based on RNA quality metrics, which were uniformly high across all samples. Following outlier removal, 27 samples were retained for subsequent analyses.

### Differential expression analysis

Differential expression analysis was performed using DESeq2 (v1.50.2), which employs a negative binomial generalized linear model and Wald statistics for hypothesis testing [40]. Genes were considered significantly differentially expressed if they met a threshold of adjusted *p*-value (Benjamini–Hochberg false discovery rate) < 0.05 and absolute log₂ fold change (|log₂FC|) > 1. Gene annotations were obtained from Ensembl using biomaRt (v2.66.0) [41].

### Functional enrichment and pathway analysis

Gene sets were obtained from the Molecular Signatures Database (MSigDB) [42–44] using the msigdbr package (v26.1.0). Mitochondrial gene sets were supplemented using the MitoCarta 3.0 database [45] and manually curated collections. Over-representation analysis (ORA) and gene set enrichment analysis (GSEA) were performed using clusterProfiler (v4.18.2) [46]. For GSEA, genes were ranked by log₂ fold change. Significance was defined as adjusted *p*-value  < 0.05. Transcription factor activity was inferred from DESeq2 results test statistic values and the decoupleR package (v2.16.0) [47] with the CollecTRI regulon database [48].

### Data visualization

Data visualization was performed using R-based tools. Heatmaps were generated from gene-wise scaled data using pheatmap (v1.0.12), and GSEA plots were generated using enrichplot (v1.30.5). Additional figures, including volcano plots and PCA, were created using ggplot2 (v4.0.2).

## Results

### TRE serum induces distinct transcriptional signatures in endothelial cells

**Fig. 1:**
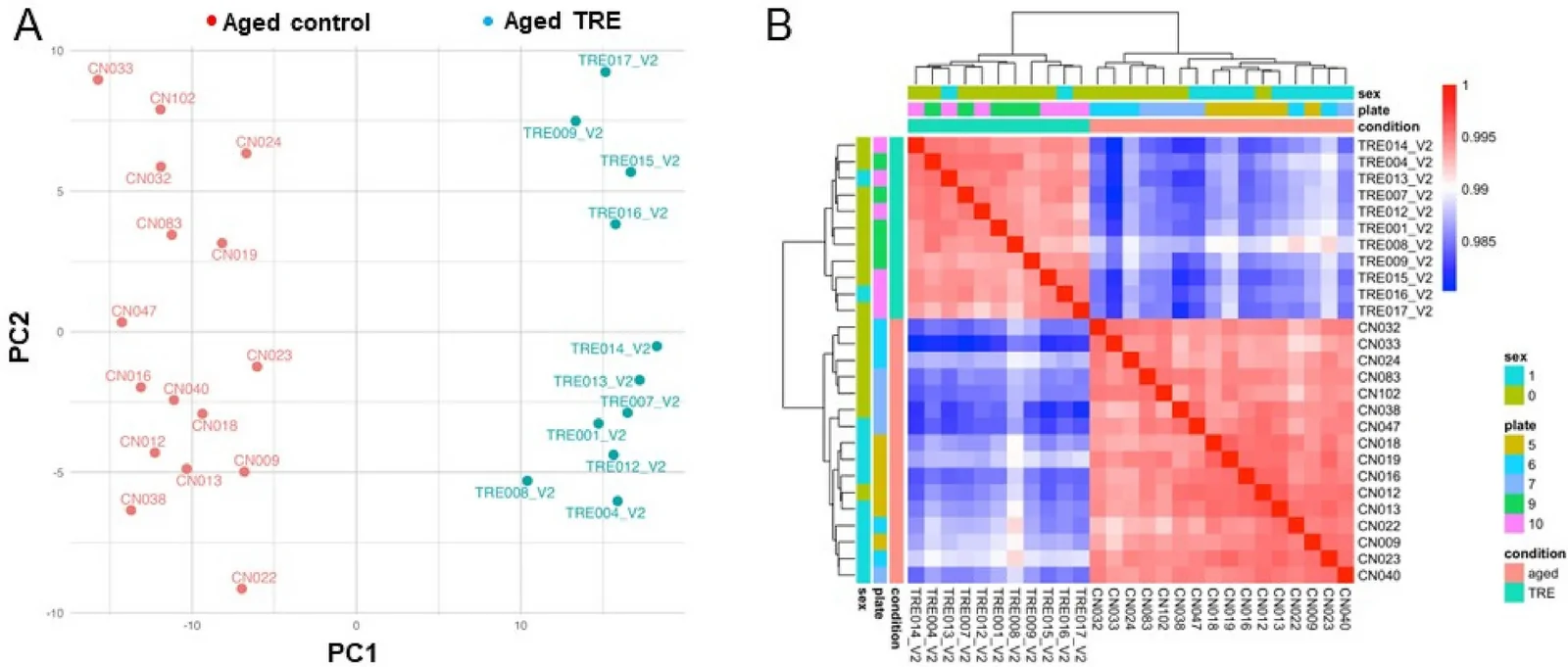
TRE serum induces distinct and reproducible transcriptional signatures in endothelial cells. **A** Principal component analysis (PCA) of gene expression profiles in hCMEC/D3 endothelial cells following 48 h treatment with aged control serum (red) or time-restricted eating (TRE) serum (blue). Samples segregate clearly along the principal components, with minimal overlap between groups, indicating that TRE serum induces a robust and consistent transcriptional reprogramming. **B** Sample-to-sample correlation heatmap with hierarchical clustering based on Pearson correlation coefficients. High within-group similarity and clear separation between TRE-treated and control samples are observed. Annotation bars indicate biological sex, sequencing plate, and treatment condition. Clustering is primarily driven by treatment condition rather than technical variables, demonstrating minimal batch effects and high reproducibility of the dataset

Principal component analysis (PCA) revealed a clear separation between endothelial cells treated with aged control serum and those treated with time-restricted eating (TRE) serum, with minimal overlap between groups (Fig. 1A). This indicates that exposure to TRE serum induces a robust and consistent transcriptional reprogramming of endothelial cells.

Sample-to-sample correlation analysis further confirmed the integrity of the dataset, demonstrating high within-group similarity and clear segregation between TRE-treated and control samples (Fig. 1B). Notably, clustering was driven primarily by treatment condition rather than technical variables such as plate or sex, indicating minimal batch effects and high reproducibility of the observed transcriptional responses.

### TRE serum induces widespread transcriptional reprogramming and activation of adaptive signaling pathways in endothelial cells

**Fig. 2:**
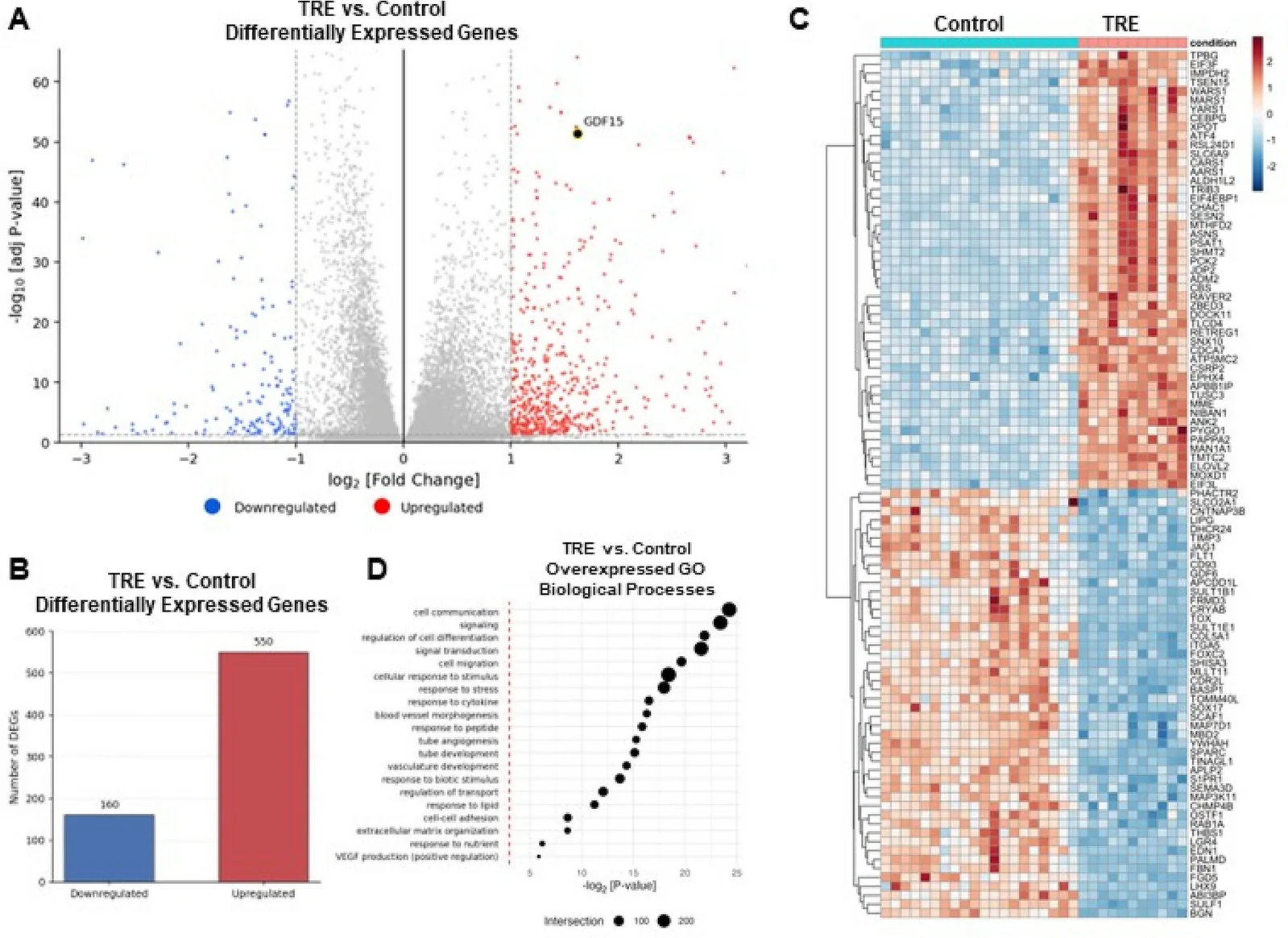
TRE serum induces widespread transcriptional reprogramming in endothelial cells. **A** Volcano plot showing differentially expressed genes (DEGs) in hCMEC/D3 endothelial cells following treatment with TRE serum compared to aged control serum. Genes are plotted by log₂ fold change and –log₁₀ adjusted *p*-value. Significantly upregulated genes are shown in red and downregulated genes in blue (adjusted *p*-value  < 0.05, |log₂FC|> 1). The stress-responsive gene *GDF15* is highlighted as a representative strongly induced transcript. **B** Summary of the number of significantly differentially expressed genes, demonstrating a predominance of upregulated transcripts in response to TRE serum. **C** Heatmap of the top differentially expressed genes with hierarchical clustering. Samples cluster according to treatment condition (control vs. TRE), with distinct transcriptional profiles characterized by coordinated upregulation (red) and downregulation (blue) of gene sets, indicating a robust and consistent treatment effect. **D** Gene Ontology (GO) biological process enrichment analysis of upregulated genes. The most significantly enriched pathways include cell communication, signaling, response to stimulus, angiogenesis-related processes, and vascular development, suggesting activation of coordinated stress-responsive and adaptive signaling programs in endothelial cells exposed to TRE serum

Differential expression analysis revealed a robust transcriptional response in endothelial cells exposed to TRE serum compared to aged control serum. Volcano plot visualization demonstrated a substantial number of significantly regulated genes, with a clear predominance of upregulated transcripts (Fig. 2A). Quantitative analysis confirmed this asymmetry, with a markedly higher number of upregulated genes relative to downregulated genes (Fig. 2B), indicating that TRE serum predominantly induces transcriptional activation rather than global suppression.

Unsupervised hierarchical clustering of the top differentially expressed genes further demonstrated clear segregation between TRE-treated and control samples (Fig. 2C). TRE-treated cells exhibited coordinated upregulation of a distinct gene cluster, accompanied by downregulation of a separate gene set, reflecting a consistent and treatment-driven transcriptional program across samples.

To gain insight into the biological processes underlying these transcriptional changes, Gene Ontology (GO) overrepresentation analysis was performed on upregulated DEGs. This analysis identified significant enrichment of pathways related to cell communication, signaling, response to stimulus, and vascular-related processes, including angiogenesis and blood vessel development (Fig. 2D). Notably, many of these pathways are consistent with activation of stress-responsive and adaptive signaling networks rather than classical pro-inflammatory activation. Collectively, these results indicate that circulating factors present in TRE serum induce a coordinated transcriptional program in endothelial cells characterized by activation of adaptive stress responses, signaling pathways, and vascular remodeling processes.

To further characterize the transcriptional programs suppressed by TRE serum, we performed overrepresentation analysis of downregulated genes. This analysis revealed coordinated downregulation of multiple core cellular processes, including pathways involved in protein synthesis, RNA processing, proteostasis, cytoskeletal organization, and growth factor signaling (Table 1).

**Table 1:** Selected downregulated biological processes in endothelial cells treated with TRE serum

Functional module	Representative pathways (GO terms)	Direction	Interpretation
Translation/ribosome biogenesis	Ribosome, rRNA processing, translational initiation	↓	Suppressed protein synthesis
RNA processing/spliceosome	mRNA splicing, spliceosome complex	↓	Reduced RNA maturation
Proteostasis	Proteasome, protein folding, chaperones	↓	Reduced protein turnover
Cell cycle/cytoskeleton	Spindle organization, microtubules, chromatid segregation	↓	Reduced proliferation/structural remodeling
Growth factor signaling	TGF-β signaling, SMAD pathways	↓	Suppressed growth signaling
Adhesion/structure	Focal adhesion, adherens junction	↓	Reduced structural programs

Specifically, gene sets related to ribosome biogenesis and translational initiation were significantly reduced, consistent with suppression of global protein synthesis. In parallel, pathways involved in mRNA processing and spliceosome function were downregulated, suggesting reduced RNA maturation capacity. Components of proteostasis networks, including proteasomal and chaperone systems, were also diminished, indicating decreased protein turnover.

Additionally, pathways governing cell cycle progression and cytoskeletal organization were suppressed, alongside reduced expression of genes involved in focal adhesion and structural integrity. Growth factor signaling pathways, including TGF-β/SMAD-associated programs, were similarly downregulated. Collectively, these findings indicate that TRE serum induces a broad suppression of anabolic and structural cellular programs, consistent with a shift toward a metabolically restrained, stress-adaptive endothelial state. This pattern is consistent with coordinated suppression of energy-intensive processes downstream of integrated stress response activation.

### TRE serum activates integrated stress response pathways and suppresses mTORC1 signaling in endothelial cells

**Fig. 3:**
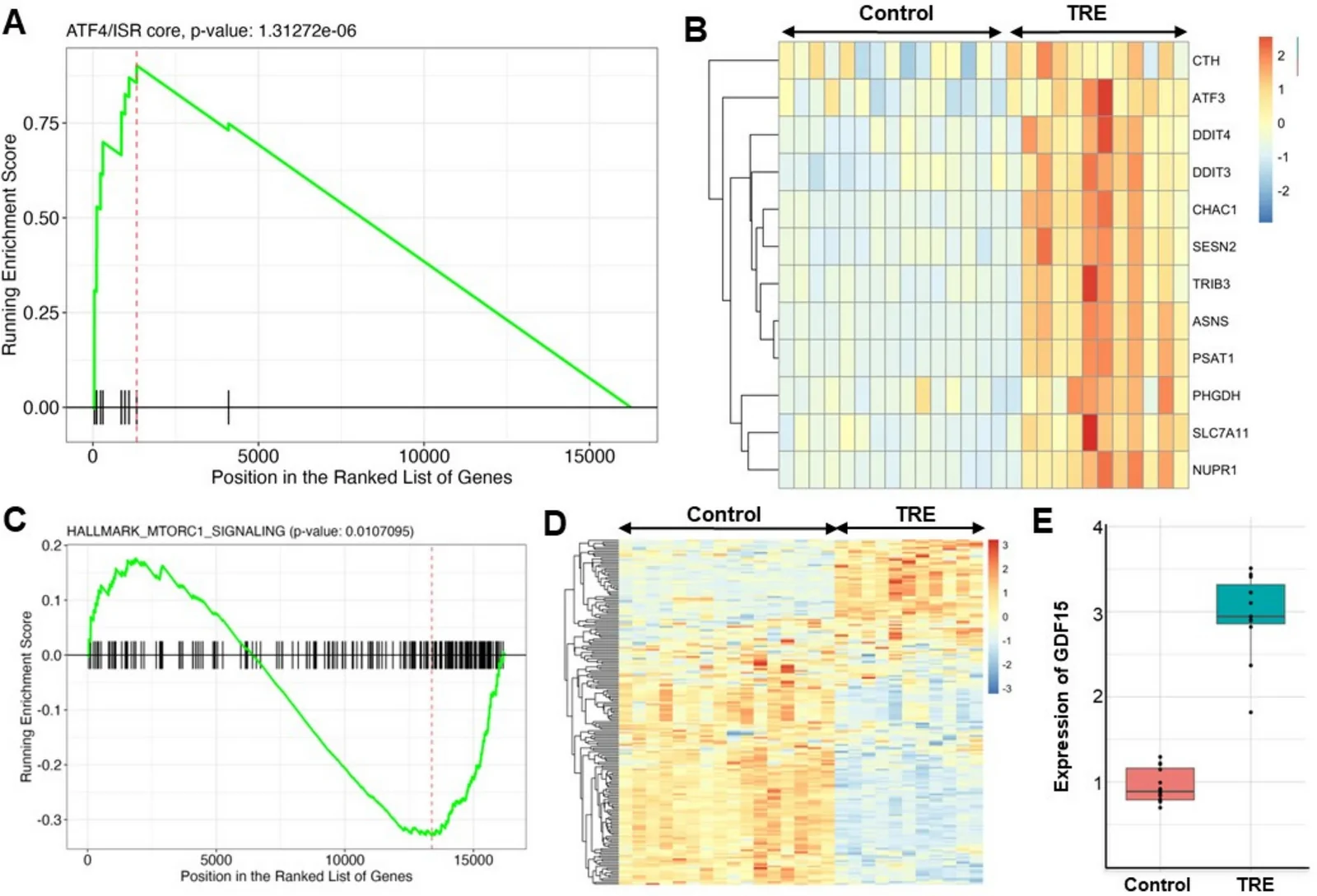
TRE serum activates integrated stress response pathways and induces *GDF15* expression in endothelial cells. **A** Gene set enrichment analysis (GSEA) demonstrating significant enrichment of the integrated stress response (ISR)/ATF4 gene signature in endothelial cells treated with TRE serum compared to control (*p* = 1.31 × 10⁻⁶). The enrichment profile indicates coordinated upregulation of ISR-associated genes. **B** Heatmap of representative ISR/ATF4 target genes, including *ATF3*, *DDIT3*, *CHAC1*, *ASNS*, *PSAT1*, and *PHGDH*, showing consistent upregulation in TRE-treated samples relative to controls. Hierarchical clustering reveals a coherent transcriptional program across samples. **C** GSEA of the HALLMARK_MTORC1_SIGNALING gene set, demonstrating significant negative enrichment (*p* = 0.0107) in TRE-treated cells, indicating suppression of mTORC1 signaling consistent with nutrient stress responses. **D** Heatmap of mTORC1-associated genes illustrating coordinated downregulation in TRE-treated endothelial cells compared to controls **E** Expression of GDF15, a stress-responsive cytokine and downstream effector of integrated stress signaling, is significantly increased in TRE-treated cells, supporting activation of adaptive metabolic stress pathways

To further define the biological programs underlying the transcriptional response to TRE serum, we performed gene set enrichment analysis (GSEA) focusing on nutrient-sensing and stress-responsive pathways. This analysis revealed a significant enrichment of the integrated stress response (ISR)/ATF4 gene signature in TRE-treated endothelial cells compared to controls (Fig. 3A), indicating activation of a coordinated transcriptional program associated with amino acid sensing and cellular adaptation to metabolic stress.

Consistent with this finding, expression analysis of canonical ISR target genes demonstrated robust and uniform upregulation of multiple ATF4-responsive transcripts, including *ATF3*, *DDIT3* (*CHOP*), *CHAC1*, *ASNS*, *PSAT1*, and *PHGDH* (Fig. 3B). Hierarchical clustering of these genes clearly segregated TRE-treated samples from controls, further supporting the activation of a conserved ISR program across biological replicates.

Given the well-established interplay between the ISR and nutrient-sensing pathways, we next examined mTORC1 signaling. GSEA revealed significant negative enrichment of the mTORC1 signaling gene set in TRE-treated cells (Fig. 3C), indicating suppression of anabolic signaling pathways typically associated with nutrient abundance. This was further supported by coordinated downregulation of mTORC1-associated genes (Fig. 3D), consistent with a shift toward a catabolic, stress-adaptive cellular state.

Importantly, these transcriptional changes were accompanied by a marked induction of *GDF15*, a stress-responsive cytokine and downstream effector of integrated stress signaling (Fig. 3E). The robust upregulation of *GDF15* provides functional support for activation of a systemic stress adaptation program and is consistent with its established role as a circulating mediator of fasting and mitochondrial stress responses. These findings demonstrate that circulating factors present in TRE serum induce a coordinated nutrient stress response in endothelial cells, characterized by activation of the ATF4/ISR axis, suppression of mTORC1 signaling, and induction of *GDF15*. This transcriptional program is consistent with a hormetic adaptation to altered metabolic conditions rather than a pathogenic stress response.

An important question raised by these findings is whether activation of the ISR reflects beneficial adaptive hormesis or a maladaptive cellular stress response [49, 50]. Several observations favor the former interpretation [51, 52]. First, ISR activation occurred in conjunction with suppression of mTORC1 signaling and enrichment of SIRT1-associated pathways, molecular adaptations that are widely recognized as components of beneficial responses to fasting and caloric restriction [34, 53]. Second, the transcriptional response was not accompanied by induction of overt injury-associated or inflammatory gene programs [54]. Third, the observed pattern was associated with enhanced expression of mitochondrial oxidative phosphorylation genes rather than transcriptional signatures indicative of mitochondrial dysfunction [52, 55]. Collectively, these findings suggest that TRE-associated circulating factors engage a coordinated adaptive stress response that promotes cellular resilience, although future functional studies will be required to distinguish adaptive from maladaptive consequences with certainty [56].

To identify upstream regulatory mechanisms coordinating these transcriptional responses, we next performed transcription factor activity analysis.

### Upstream transcriptional analysis identifies coordinated stress and metabolic regulatory networks induced by TRE serum

To identify upstream regulatory mechanisms underlying the transcriptional reprogramming induced by TRE serum, we performed transcription factor (TF) activity inference using a regulon-based approach (Table 2). This analysis revealed coordinated activation of multiple transcriptional networks associated with cellular stress responses, metabolic adaptation, and immune signaling.

**Table 2:** Predicted upstream transcriptional regulators and functional modules induced by TRE serum

Functional module	Transcriptional regulators	Direction	Interpretation
Integrated stress response (ISR)/metabolic adaptation	ATF4, FOXO1, FOXO3, KLF15	↑ Activated	Nutrient stress adaptation, amino acid sensing, metabolic reprogramming
Interferon/innate immune signaling	IRF1, IRF2, IRF9, STAT1, STAT2	↑ Activated	Innate immune-like signaling, consistent with stress-induced transcriptional programs
NF-κB-associated signaling	NFKB1, REL, RELA	↑ Activated	Stress-responsive transcriptional regulation; likely reflects adaptive signaling rather than classical inflammatory activation
Antigen presentation (MHC II regulation)	CIITA, RFXANK, RFX5	↑ Activated	Enhanced immune surveillance/antigen processing pathways
Lipid and cholesterol metabolism	SREBF1, SREBF2	↓ Inhibited	Suppression of anabolic lipid biosynthesis consistent with mTORC1 inhibition
Endothelial homeostasis/quiescence	HOPX	↓ Inhibited	Shift from quiescent toward a more responsive/adaptive endothelial state
Genomic stability/stress response	BRCA1, HMGB2, ABL1	↑ Activated	DNA damage response and cellular stress adaptation mechanisms

Among the most prominently activated regulators were members of the interferon regulatory factor and JAK–STAT families, including *IRF1*, *IRF2*, *IRF9*, *STAT1*, and *STAT2*, consistent with enrichment of innate immune and stimulus-responsive transcriptional programs. In parallel, components of the NF-κB pathway were also predicted to be activated. Notably, these TFs are known to be responsive to redox and metabolic stress, suggesting that their activation reflects adaptive signaling processes rather than classical pro-inflammatory endothelial activation.

Importantly, transcription factors associated with the integrated stress response (ISR) and metabolic adaptation were also enriched, including *ATF4*,* FOXO1*, *FOXO3*, and *KLF15*. These findings are highly consistent with the GSEA results demonstrating activation of ATF4/ISR pathways and support the presence of a coordinated nutrient stress response in TRE-treated endothelial cells.

In contrast, key regulators of anabolic metabolism were predicted to be inhibited, including *SREBF1* and *SREBF2*, which are central drivers of lipid and cholesterol biosynthesis. This observation is in line with the suppression of mTORC1 signaling and further supports a shift toward a catabolic, nutrient-adaptive cellular state. Additionally, the endothelial quiescence-associated factor *HOPX* was predicted to be inhibited, suggesting a transition from a quiescent phenotype toward a more dynamically responsive state. These findings indicate that time-restricted eating (TRE) serum induces a coordinated upstream regulatory program characterized by activation of stress-responsive and metabolic transcriptional networks, coupled with suppression of anabolic pathways. This regulatory architecture is consistent with a hormetic adaptation to altered nutrient availability, integrating integrated stress response (ISR) activation, metabolic reprogramming, and selective engagement of stress-responsive signaling pathways. Together, these data position transcriptional regulation as a central integrator of endothelial responses to circulating factors induced by time-restricted eating.

### TRE serum promotes mitochondrial functional remodeling without induction of canonical biogenesis programs

**Fig. 4:**
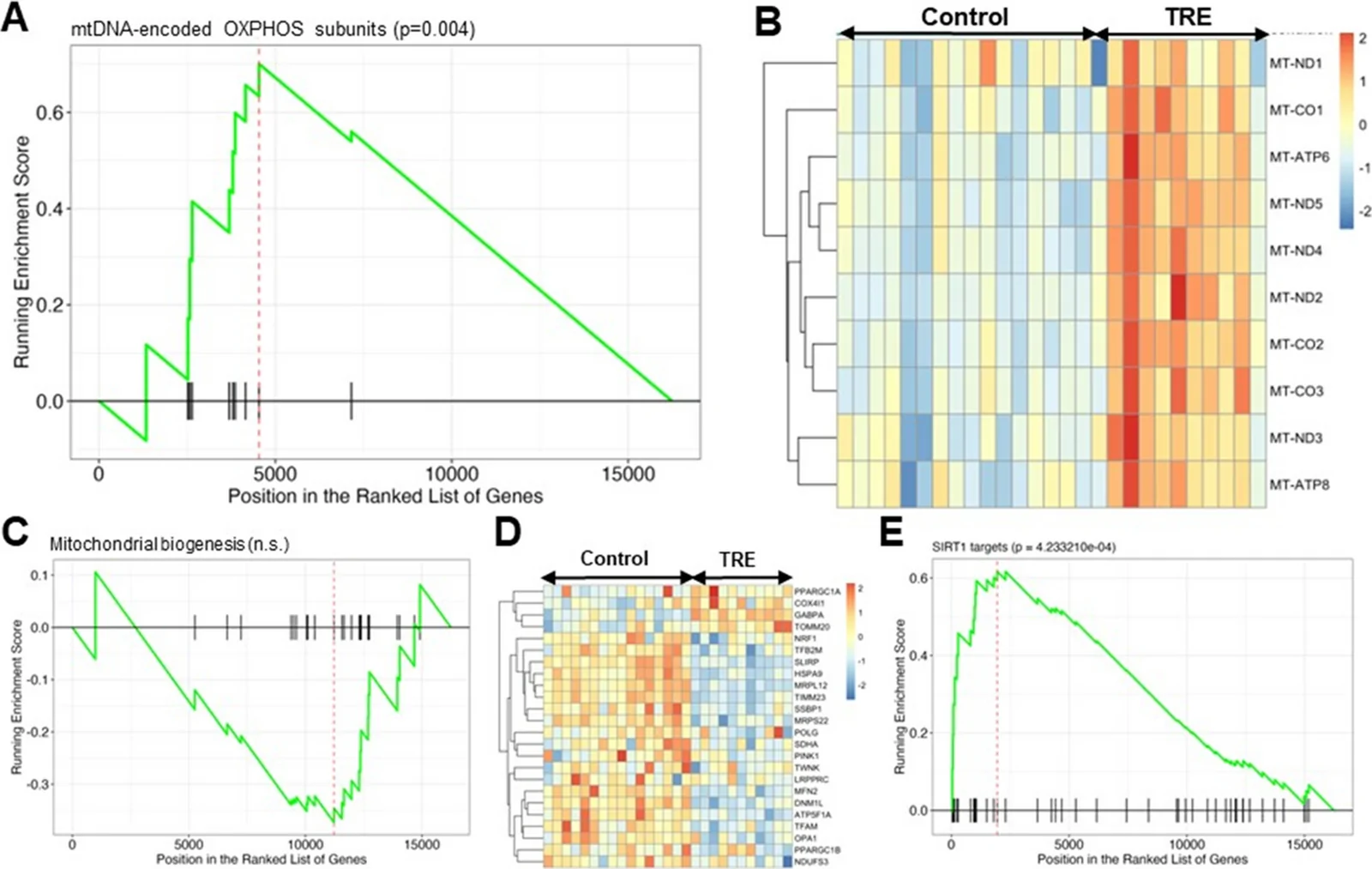
TRE serum induces mitochondrial functional remodeling without activation of canonical mitochondrial biogenesis pathways. **A** Gene set enrichment analysis (GSEA) of mitochondrial DNA (mtDNA)-encoded oxidative phosphorylation (OXPHOS) subunits demonstrates significant positive enrichment in TRE-treated endothelial cells compared to controls (*p* = 0.004), indicating increased expression of core mitochondrial respiratory chain components. **B** Heatmap of mtDNA-encoded OXPHOS genes (*MT-ND1–ND6*, *MT-CO1–CO3*, *MT-ATP6*, *MT-ATP8*) showing consistent upregulation across TRE-treated samples relative to controls. **C** GSEA of a mitochondrial biogenesis gene set reveals no significant enrichment (n.s.), indicating that transcriptional activation of canonical mitochondrial biogenesis pathways is not induced by TRE serum. **D** Heatmap of nuclear-encoded mitochondrial biogenesis regulators (including *PPARGC1A, NRF1, TFAM,* and related genes) showing heterogeneous expression patterns without coordinated upregulation in TRE-treated cells. **(E)** GSEA of SIRT1 target genes demonstrates significant positive enrichment in TRE-treated endothelial cells (*p* = 4.23 × 10⁻^4^), consistent with activation of nutrient-sensing and metabolic adaptation pathways

Given the activation of integrated stress response pathways in TRE-treated endothelial cells, we next examined whether these changes were associated with alterations in mitochondrial biology. GSEA revealed significant positive enrichment of mitochondrial DNA (mtDNA)-encoded oxidative phosphorylation (OXPHOS) subunits in TRE-treated cells compared to controls (Fig. 4A), indicating increased expression of core components of the mitochondrial respiratory chain. Consistent with this finding, heatmap analysis demonstrated coordinated upregulation of mtDNA-encoded genes, including *MT-ND1–ND6*, *MT-CO1–CO3*, and *MT-ATP6/8*, across TRE-treated samples (Fig. 4B). These results suggest enhanced mitochondrial functional capacity at the level of electron transport chain components.

To determine whether this transcriptional response reflected activation of mitochondrial biogenesis, we performed GSEA using a curated mitochondrial biogenesis gene set. In contrast to the robust enrichment observed for mtDNA-encoded genes, mitochondrial biogenesis pathways were not significantly enriched (Fig. 4C). Analysis of nuclear-encoded regulators of mitochondrial biogenesis, including *PPARGC1A* (*PGC-1α*),* NRF1*, and *TFAM*, further revealed heterogeneous expression patterns without coordinated upregulation in TRE-treated cells (Fig. 4D), arguing against induction of canonical biogenesis programs.

Given the established role of SIRT1 in mediating metabolic adaptations to nutrient availability, we next assessed SIRT1-associated transcriptional programs. GSEA demonstrated significant enrichment of SIRT1 target genes in TRE-treated endothelial cells (Fig. 4E), consistent with activation of nutrient-sensing pathways linked to fasting-like states. Collectively, these findings indicate that TRE serum induces a distinct mitochondrial transcriptional phenotype characterized by increased expression of respiratory chain components and activation of SIRT1-associated metabolic programs, without concurrent activation of canonical mitochondrial biogenesis pathways. This pattern suggests a shift toward mitochondrial functional optimization rather than expansion of mitochondrial mass, consistent with adaptive responses to altered nutrient availability.

### Lack of coordinated activation of autophagy and BBB maintenance pathways

**Fig. 5:**
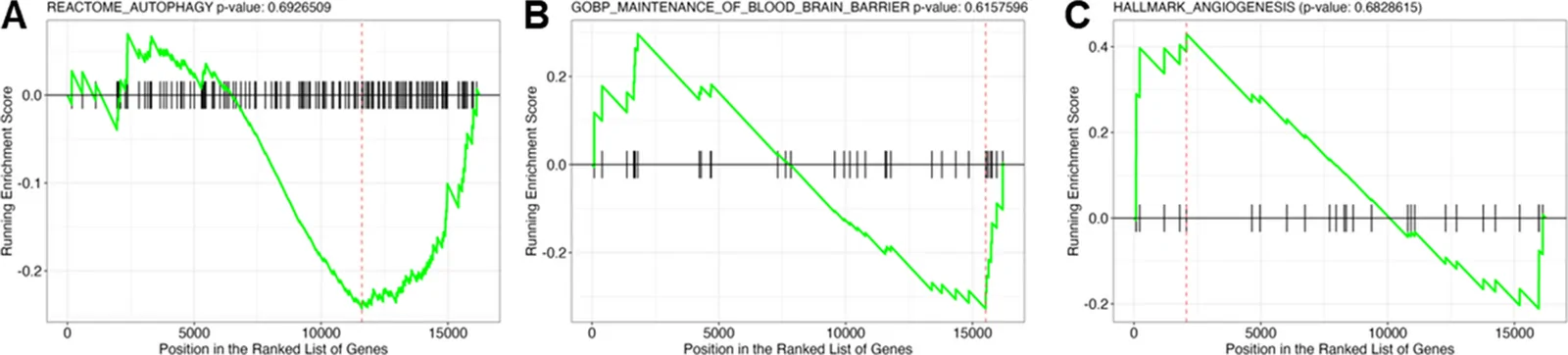
TRE serum does not significantly alter autophagy, blood–brain barrier maintenance, or angiogenesis gene programs in endothelial cells. **A** Gene set enrichment analysis (GSEA) of the REACTOME_AUTOPHAGY pathway shows no significant enrichment in endothelial cells treated with TRE serum compared to control (*p* = 0.69), indicating that canonical autophagy-related transcriptional programs are not substantially altered. **B** GSEA of the Gene Ontology biological process “maintenance of blood–brain barrier” (GOBP_MAINTENANCE_OF_BLOOD_BRAIN_BARRIER) demonstrates no significant enrichment (*p* = 0.62), suggesting that core transcriptional programs associated with blood–brain barrier integrity are not significantly modulated by TRE serum. **C** GSEA of the HALLMARK_ANGIOGENESIS gene set reveals no significant enrichment (*p* = 0.68), indicating that angiogenic transcriptional programs are not robustly activated or suppressed under these conditions

In parallel, GSEA revealed no significant enrichment of pathways related to autophagy or blood–brain barrier (BBB) maintenance (Fig. 5). The REACTOME autophagy gene set was not significantly enriched (*p* = 0.693), with pathway-associated genes broadly dispersed across the ranked list and no consistent directional shift in the running enrichment score, indicating the absence of coordinated transcriptional regulation. Similarly, a gene set associated with BBB maintenance did not reach significance (*p* = 0.616), with an enrichment profile suggestive of a lack of synchronized expression of BBB-supportive genes. Collectively, these findings demonstrate that TRE serum does not elicit coordinated transcriptional activation of autophagy or BBB maintenance programs. Instead, the response appears to involve selective modulation of individual genes rather than engagement of canonical pathway-level programs.

### TRE serum induces angiogenic signaling components without activating a coordinated angiogenic program

Despite the upregulation of individual angiogenesis-associated genes, including VEGFA, exposure to TRE serum did not result in the activation of a coordinated angiogenic transcriptional program. Instead, the gene expression profile was characterized by selective modulation of vascular signaling pathways, including increased expression of matrix-remodeling and signaling-related factors, alongside relative suppression or lack of induction of canonical developmental angiogenic drivers.

Consistent with this pattern, gene set enrichment analysis (GSEA) demonstrated that the HALLMARK angiogenesis gene set (MSigDB) was not significantly enriched (*p* = 0.683). Although the running enrichment score exhibited an early positive peak—indicating partial representation of angiogenesis-related genes among the top-ranked genes—this signal was not sustained across the ranked gene list, with the enrichment curve shifting toward negative values in later positions (Fig. 5C). This pattern indicates that angiogenesis-related genes are not coordinately regulated but rather display a fragmented and non-synchronous expression profile. Together, these findings suggest that TRE serum promotes a state of endothelial signaling plasticity and vascular remodeling, rather than inducing a full angiogenic program.

## Discussion

Vascular cognitive impairment and dementia (VCID) are increasingly recognized as major contributors to unhealthy brain aging, with cerebral microcirculatory dysfunction emerging as a central pathogenic mechanism [2–5]. In this study, we demonstrate that circulating factors derived from humans practicing TRE induce a robust and coordinated transcriptional reprogramming in human cerebromicrovascular endothelial cells. This response is characterized by activation of stress-adaptive and metabolic pathways, suppression of anabolic programs, and remodeling of mitochondrial function. Together, these findings provide new insight into the systemic mechanisms by which TRE may influence vascular aging and support the concept that circulating factors act as key mediators of its biological effects.

A central finding of this study is the activation of the integrated stress response (ISR) [57, 58] and concomitant suppression of mTORC1 signaling [59] in TRE-treated endothelial cells. The ISR represents a conserved adaptive program that enables cells to respond to nutrient limitation, amino acid imbalance, and other forms of metabolic stress by reprogramming transcriptional and translational processes. Activation of ATF4-dependent transcription, together with suppression of mTORC1 signaling, is consistent with a shift away from anabolic growth toward a catabolic, stress-adaptive state [60]. This pattern aligns closely with known cellular responses to fasting and caloric restriction and supports the notion that TRE engages conserved nutrient-sensing pathways at the level of the endothelium.

Importantly, this transcriptional reprogramming was accompanied by robust induction of *GDF15*, a stress-responsive cytokine increasingly recognized as a systemic mediator of metabolic adaptation [61–63]. Elevated *GDF15* has been linked to mitochondrial stress, nutrient deprivation, and lifespan-extending interventions, and is thought to act as a circulating signal coordinating organismal responses to metabolic challenges [61–65]. Its induction in endothelial cells exposed to TRE serum provides further evidence that circulating factors associated with TRE engage conserved stress-adaptive pathways and suggests a potential mechanism through which systemic metabolic states may influence vascular function [66, 67]. Although GDF15 is commonly viewed as a biomarker of mitochondrial stress and ISR activation, accumulating evidence suggests that it may also function as an active regulator of metabolic adaptation [58, 68–70]. Experimental studies have implicated GDF15 in the regulation of energy balance, mitochondrial homeostasis, angiogenesis, and tissue stress responses. Thus, the marked induction of GDF15 observed in TRE serum-treated endothelial cells may not merely reflect activation of adaptive stress pathways but could also contribute to downstream vascular adaptations. Further studies will be required to determine whether GDF15 acts as a mediator of the endothelial effects induced by TRE-associated circulating factors [71, 72].

At the mitochondrial level, TRE serum induced a distinct transcriptional phenotype characterized by increased expression of mitochondrial DNA–encoded oxidative phosphorylation components, without concomitant activation of canonical mitochondrial biogenesis pathways. This dissociation suggests that TRE-associated circulating factors promote mitochondrial functional remodeling rather than expansion of mitochondrial mass. Such a pattern is consistent with enhanced mitochondrial efficiency, a feature commonly associated with metabolic interventions that extend healthspan [73]. The concurrent enrichment of SIRT1-associated transcriptional programs further supports activation of nutrient-sensing pathways linked to metabolic flexibility and mitochondrial regulation. Notably, this interpretation is supported by ongoing work from our group demonstrating that time-restricted feeding in mice similarly enhances vascular mitochondrial respiration and oxidative phosphorylation capacity [14] and is associated with upregulation of the endothelial mitochondrial transcriptome in vivo (manuscript submitted). Together, these observations suggest that TRE-associated circulating factors may recapitulate conserved metabolic adaptations across experimental systems [74].

An intriguing aspect of these findings is the dissociation between increased expression of oxidative phosphorylation genes and the absence of coordinated activation of canonical mitochondrial biogenesis pathways. This pattern suggests that TRE-associated circulating factors, similar to caloric restriction [75, 76], may promote mitochondrial functional optimization rather than expansion of mitochondrial mass. Such remodeling could reflect improved respiratory efficiency, enhanced mitonuclear coordination [77], or adaptive metabolic reprogramming associated with fasting-induced hormesis. Similar patterns have been reported in other longevity-promoting dietary interventions and may represent a common mechanism through which metabolic interventions improve cellular resilience [75–78].

A notable aspect of our findings is the coordinated suppression of anabolic and structural cellular programs, including pathways related to protein synthesis, RNA processing [79], proteostasis, cytoskeletal organization, and growth factor signaling [34, 80, 81]. These changes are consistent with a global reduction in energy-intensive processes and further support the interpretation that TRE serum induces a metabolically restrained, stress-adaptive endothelial phenotype. From a geroscience perspective, such suppression of growth-related pathways may represent a protective mechanism that enhances cellular resilience and reduces susceptibility to damage.

Transcription factor analysis revealed that these changes are orchestrated by a coordinated regulatory network integrating stress-responsive and metabolic signaling pathways. Activation of ATF4, FOXO, and KLF family transcription factors is consistent with ISR activation and metabolic adaptation, while inhibition of SREBF1/2 aligns with suppression of lipid biosynthesis and mTORC1 signaling [50, 82–96]. Interestingly, transcriptional programs associated with interferon signaling and NF-κB were also enriched. While these pathways are classically associated with inflammation, they are also known to be activated by redox and metabolic stress and to participate in adaptive cellular responses. Importantly, canonical markers of endothelial activation were not prominently represented among the most strongly induced transcripts, suggesting that these signals reflect a stress-responsive, hormetic adaptation rather than a pathogenic inflammatory phenotype.

An important finding of this study is the absence of coordinated activation of canonical endothelial functional programs such as autophagy, and BBB maintenance. Despite modest regulation of individual genes within these pathways, gene set enrichment analyses did not reveal sustained or coordinated transcriptional activation. This suggests that the endothelial response to TRE-associated circulating factors is selective and does not involve broad activation of vascular remodeling or repair programs at the transcriptional level. Interestingly, individual angiogenesis-associated genes, including VEGFA, were modestly induced, suggesting selective enhancement of endothelial signaling plasticity without activation of a coordinated angiogenic program. Instead, the dominant response appears to involve metabolic and stress-adaptive reprogramming. The absence of coordinated transcriptional activation of autophagy-related pathways was somewhat unexpected given the well-established links between fasting, mTOR inhibition, and autophagic responses [11, 81]. Several explanations are possible. Autophagy is regulated extensively at post-transcriptional and post-translational levels and therefore may not be adequately captured by transcriptomic analyses alone. In addition, autophagic activation may occur transiently and have resolved by the time endothelial cells were harvested after 48 h of serum exposure. Finally, fasting-induced autophagy responses may exhibit substantial cell-type specificity. Future studies examining autophagic flux directly will be necessary to determine whether TRE-associated circulating factors modulate autophagy in cerebromicrovascular endothelial cells.

Our findings are consistent with and extend previous work demonstrating that circulating factors mediate many of the beneficial effects of dietary interventions. Prior studies by de Cabo and colleagues and by our laboratories have shown that serum derived from CR animals confers endothelial protection and enhances stress resistance in vitro [25–28, 30, 31]. Our data suggest that similar principles may apply to TRE in humans, supporting the concept that circulating factors represent a key interface between systemic metabolic state and vascular function. The identity of the circulating factors responsible for the observed endothelial responses remains unknown [97]. Several candidate mediators warrant consideration [11]. Time-restricted eating has been associated with alterations in circulating ketone bodies [98], amino acid profiles, insulin and IGF-1 signaling, bile acids [99], adipokines, and fasting-associated hormones such as FGF21 [100]. Each of these factors has been implicated in the regulation of nutrient sensing, mitochondrial function, and endothelial biology. It is therefore plausible that the transcriptional response observed in the present study reflects the integrated actions of multiple circulating mediators rather than the effects of a single factor. Identifying these circulating signals represents an important objective for future mechanistic studies. Importantly, while TRE does not necessarily reduce caloric intake, it appears to engage overlapping nutrient-sensing and stress-response pathways, leading to comparable downstream effects.

Our findings further support the emerging concept that vascular aging is regulated not exclusively through cell-autonomous mechanisms, but also through circulating systemic signals capable of reprogramming endothelial phenotypes [19, 97]. In this context, the present study provides additional evidence that age- and diet-dependent alterations in the systemic milieu can directly modulate cerebromicrovascular endothelial transcriptional programs [19]. These observations are consistent with growing evidence from heterochronic parabiosis and plasma transfer studies demonstrating an important role for circulating pro-geronic and anti-geronic factors in the regulation of vascular aging and endothelial resilience [19].

From a translational perspective, these findings have important implications for VCID and vascular aging. Endothelial dysfunction in aging is characterized not only by impaired signaling but also by reduced adaptive capacity [101–107]. The ability of TRE-associated circulating factors to induce a coordinated stress-adaptive program suggests a potential mechanism by which this intervention could enhance endothelial resilience and preserve microvascular function [11, 14]. This is particularly relevant for the cerebral microcirculation, where maintenance of endothelial function is critical for neurovascular coupling and cognitive health [7, 108].

From a vascular aging perspective, the transcriptional adaptations induced by TRE-associated serum are noteworthy because they converge on pathways known to influence endothelial resilience. Activation of ISR/ATF4 signaling, suppression of mTORC1 activity, induction of SIRT1-associated programs, and remodeling of mitochondrial function have each been linked to improved cellular stress resistance and preservation of vascular homeostasis. Although the present study was not designed to evaluate functional outcomes directly, these molecular adaptations would be predicted to enhance resistance to oxidative and metabolic stressors that contribute to age-related cerebromicrovascular dysfunction and VCID. Future studies should therefore prioritize assessment of endothelial mitochondrial respiration, nitric oxide bioavailability, barrier integrity, angiogenic competence, oxidative stress resistance, and neurovascular coupling-related functions to establish the physiological significance of the transcriptional responses identified here.

Several limitations should be considered. First, the study employs an in vitro serum transfer model, which, while powerful for dissecting cell-autonomous responses to circulating factors, does not capture the full complexity of in vivo physiology. Second, transcriptomic changes do not necessarily translate directly into functional outcomes, and additional studies are needed to link these molecular signatures to endothelial function. Third, the identity of the specific circulating mediators responsible for these effects remains to be determined. Future studies should aim to identify the molecular components of TRE-associated serum responsible for these effects and to validate their functional impact using in vivo models. Integration of transcriptomic, proteomic, and metabolomic approaches will be critical to fully understand how systemic metabolic interventions reshape the circulating milieu and influence vascular aging. An additional limitation of the study is the limited availability of donor-level clinical metadata. Future studies incorporating comprehensive donor phenotyping will be important for determining which clinical and metabolic characteristics are most strongly associated with the endothelial responses induced by TRE-associated circulating factors.

In conclusion, our findings demonstrate that circulating factors induced by time-restricted eating drive a coordinated endothelial response characterized by activation of stress-adaptive and metabolic pathways, suppression of anabolic processes, and mitochondrial remodeling. These results support a model in which TRE promotes vascular health not through direct activation of classical repair pathways, but through induction of a hormetic, metabolically optimized endothelial state. This framework provides new insight into the systemic regulation of vascular aging and highlights circulating factors as key mediators linking lifestyle interventions to cerebrovascular health.

## References

[1] Major D, Dósa N, Balázs P, Fekete M, Pártos K, Árva D, et al. Global trends in the incidence and prevalence of Alzheimer’s disease. Adv Trans Res. 2026;1:21–8. 10.1556/1661.2025.00106.

[2] Iadecola C, Duering M, Hachinski V, Joutel A, Pendlebury ST, Schneider JA, et al. Vascular cognitive impairment and dementia: JACC scientific expert panel. J Am Coll Cardiol. 2019;73:3326–44. 10.1016/j.jacc.2019.04.034. PMC6719789 31248555

[3] van der Flier WM, Skoog I, Schneider JA, Pantoni L, Mok V, Chen CLH, et al. Vascular cognitive impairment. Nat Rev Dis Primers. 2018;4:18003. 10.1038/nrdp.2018.3. 29446769

[4] Dichgans M, Leys D. Vascular cognitive impairment. Circ Res. 2017;120:573–91. 10.1161/CIRCRESAHA.116.308426. 28154105

[5] Sachdev PS, Bentvelzen AC, Gustafson D, Hansra GK, Hosoki S, Jiang J, et al. Vascular cognitive impairment and dementia: clinical features, neuropathology, and biomarkers. J Am Coll Cardiol. 2026;87:52–76. 10.1016/j.jacc.2025.11.008. PMC13553213 41498479

[6] Goodall LS, Lennon MJ, Sachdev PS, Gorelick PB, Kovacic JC, Samaras K. Current and emerging therapeutic approaches for vascular cognitive impairment and dementia. J Am Coll Cardiol. 2026;87:77–100. 10.1016/j.jacc.2025.09.1502. 41498480

[7] Toth P, Tarantini S, Csiszar A, Ungvari Z. Functional vascular contributions to cognitive impairment and dementia: mechanisms and consequences of cerebral autoregulatory dysfunction, endothelial impairment, and neurovascular uncoupling in aging. Am J Physiol Heart Circ Physiol. 2017;312:H1–20. 10.1152/ajpheart.00581.2016. PMC5283909 27793855

[8] Iadecola C, Smith EE, Anrather J, Gu C, Mishra A, Misra S, et al. The neurovasculome: key roles in brain health and cognitive impairment: a scientific statement from the American Heart Association/American Stroke Association. Stroke. 2023;54:e251–71. 10.1161/STR.0000000000000431. PMC10228567 37009740

[9] Sweeney MD, Zhao Z, Montagne A, Nelson AR, Zlokovic BV. Blood-brain barrier: from physiology to disease and back. Physiol Rev. 2019;99:21–78. 10.1152/physrev.00050.2017. PMC6335099 30280653

[10] Kisler K, Nelson AR, Montagne A, Zlokovic BV. Cerebral blood flow regulation and neurovascular dysfunction in Alzheimer disease. Nat Rev Neurosci. 2017;18:419–34. 10.1038/nrn.2017.48. PMC5759779 28515434

[11] Milan M, Troyano-Rodriguez E, Ihuoma J, Negri S, Rudraboina R, Kosmider A, et al. Fasting as medicine: mitochondrial and endothelial rejuvenation in vascular aging. Aging Cell. 2026;25:e70372. 10.1111/acel.70372. PMC12791036 41521387

[12] Gulej R, Patai R, Ungvari A, Tordai A, Huffman DM, Tarantini S, et al. Plasma-based strategies for systemic rejuvenation: critical perspectives on clinical translation. Geroscience. 2026. 10.1007/s11357-026-02136-8. PMC13356195 41721191

[13] Liberale L, Tual-Chalot S, Sedej S, Ministrini S, Georgiopoulos G, Grunewald M, et al. Roadmap for alleviating the manifestations of ageing in the cardiovascular system. Nat Rev Cardiol. 2025;22:577–605. 10.1038/s41569-025-01130-5. PMC13383118 39972009

[14] Milan M, Brown J, O’Reilly CL, Bubak MP, Negri S, Balasubramanian P, et al. Time-restricted feeding improves aortic endothelial relaxation by enhancing mitochondrial function and attenuating oxidative stress in aged mice. Redox Biol. 2024;73:103189. 10.1016/j.redox.2024.103189. PMC11140804 38788541

[15] Balasubramanian P, DelFavero J, Ungvari A, Papp M, Tarantini A, Price N, et al. Time-restricted feeding (TRF) for prevention of age-related vascular cognitive impairment and dementia. Ageing Res Rev. 2020;64:101189. 10.1016/j.arr.2020.101189. PMC7710623 32998063

[16] Negri S, Sanford M, Reyff Z, Ballard C, Nyul-Toth A, Gulej R, et al. Transcriptomic signatures of time-restricted feeding (TRF) in cerebrovascular aging and cognitive health. Vascul Pharmacol. 2024;155:107338. 10.1016/j.vph.2024.107338.

[17] Huston CA, Milan M, Vance ML, Bickel MA, Miller LR, Negri S, et al. The effects of time restricted feeding on age-related changes in the mouse retina. Exp Gerontol. 2024;194:112510. 10.1016/j.exger.2024.112510. PMC11425985 38964431

[18] Gulej R, Patai R, Chandragiri SS, Nagykaldi M, Brunner E, Mukli P, et al. Young blood-induced rejuvenation of neurovascular coupling involves endothelial IGF-1/IGF-1R signaling: evidence from heterochronic parabiosis using endothelial IGF-1R deficient and systemic IGF-1 knockdown mice. Geroscience. 2026. 10.1007/s11357-026-02118-w. PMC13356212 41714560

[19] Gulej R, Patai R, Ungvari A, Kallai A, Tarantini S, Yabluchanskiy A, et al. Impacts of systemic milieu on cerebrovascular and brain aging: insights from heterochronic parabiosis, blood exchange, and plasma transfer experiments. Geroscience. 2025;47:6207–376. 10.1007/s11357-025-01657-y. PMC12635022 40407975

[20] Gulej R, Nyul-Toth A, Csik B, Petersen B, Faakye J, Negri S, et al. Rejuvenation of cerebromicrovascular function in aged mice through heterochronic parabiosis: insights into neurovascular coupling and the impact of young blood factors. Geroscience. 2024;46:327–47. 10.1007/s11357-023-01039-2. PMC10828280 38123890

[21] Gulej R, Nyul-Toth A, Csik B, Patai R, Petersen B, Negri S, et al. Young blood-mediated cerebromicrovascular rejuvenation through heterochronic parabiosis: enhancing blood-brain barrier integrity and capillarization in the aged mouse brain. Geroscience. 2024;46:4415–42. 10.1007/s11357-024-01154-8. PMC11336025 38727872

[22] Kiss T, Nyul-Toth A, Gulej R, Tarantini S, Csipo T, Mukli P, et al. Old blood from heterochronic parabionts accelerates vascular aging in young mice: transcriptomic signature of pathologic smooth muscle remodeling. Geroscience. 2022;44:953–81. 10.1007/s11357-022-00519-1. PMC9135944 35124764

[23] Kiss T, Tarantini S, Csipo T, Balasubramanian P, Nyul-Toth A, Yabluchanskiy A, et al. Circulating anti-geronic factors from heterochonic parabionts promote vascular rejuvenation in aged mice: transcriptional footprint of mitochondrial protection, attenuation of oxidative stress, and rescue of endothelial function by young blood. Geroscience. 2020;42:727–48. 10.1007/s11357-020-00180-6. PMC7205954 32172434

[24] Razi S, Cogger VC, Kennerson M, Benson VL, McMahon AC, Blyth FM, et al. SIRT1 polymorphisms and serum-induced SIRT1 protein expression in aging and frailty: the CHAMP study. J Gerontol A Biol Sci Med Sci. 2017;72:870–6. 10.1093/gerona/glx018. PMC6279142 28329314

[25] de Cabo R, Liu L, Ali A, Price N, Zhang J, Wang M, et al. Serum from calorie-restricted animals delays senescence and extends the lifespan of normal human fibroblasts in vitro. Aging (Albany NY). 2015;7:152–66. 10.18632/aging.100719. PMC4394727 25855056

[26] Csiszar A, Labinskyy N, Jimenez R, Pinto JT, Ballabh P, Losonczy G, et al. Anti-oxidative and anti-inflammatory vasoprotective effects of caloric restriction in aging: role of circulating factors and SIRT1. Mech Ageing Dev. 2009;130:518–27. 10.1016/j.mad.2009.06.004. PMC2756526 19549533

[27] Allard JS, Heilbronn LK, Smith C, Hunt ND, Ingram DK, Ravussin E, et al. In vitro cellular adaptations of indicators of longevity in response to treatment with serum collected from humans on calorie restricted diets. PLoS ONE. 2008;3:e3211. 10.1371/journal.pone.0003211. PMC2527659 18791640

[28] Cohen HY, Miller C, Bitterman KJ, Wall NR, Hekking B, Kessler B, et al. Calorie restriction promotes mammalian cell survival by inducing the SIRT1 deacetylase. Science. 2004;305:390–2. 10.1126/science.1099196 15205477

[29] Duregon E, Fernandez ME, Martinez Romero J, Di Germanio C, Cabassa M, Voloshchuk R, et al. Prolonged fasting times reap greater geroprotective effects when combined with caloric restriction in adult female mice. Cell Metab. 2023;35:1179-1194 e1175. 10.1016/j.cmet.2023.05.003. PMC10369303 37437544

[30] Csiszar A, Sosnowska D, Tucsek Z, Gautam T, Toth P, Losonczy G, et al. Circulating factors induced by caloric restriction in the nonhuman primate *Macaca mulatta* activate angiogenic processes in endothelial cells. J Gerontol A Biol Sci Med Sci. 2013;68:235–49. 10.1093/gerona/gls158. PMC3605906 22904098

[31] de Cabo R, Furer-Galban S, Anson RM, Gilman C, Gorospe M, Lane MA. An in vitro model of caloric restriction. Exp Gerontol. 2003;38:631–9. 10.1016/s0531-5565(03)00055-x 12814798

[32] Tippairote T, Janssen S, Chunhabundit R. Restoration of metabolic tempo through time-restricted eating (TRE) as the preventive measure for metabolic diseases. Crit Rev Food Sci Nutr. 2021;61:2444–53. 10.1080/10408398.2020.1781050. 32551943

[33] Manoogian ENC, Chow LS, Taub PR, Laferrere B, Panda S. Time-restricted eating for the prevention and management of metabolic diseases. Endocr Rev. 2022;43:405–36. 10.1210/endrev/bnab027. PMC8905332 34550357

[34] de Cabo R, Mattson MP. Effects of intermittent fasting on health, aging, and disease. N Engl J Med. 2019;381:2541–51. 10.1056/NEJMra1905136. 31881139

[35] Yi X, Abas R, Raja Muhammad Rooshdi RAW, Yan J, Liu C, An J, et al. Time-restricted feeding attenuated hypertension-induced cardiac remodeling by modulating autophagy levels in spontaneously hypertensive rats. Sci Rep. 2025;15:16973. 10.1038/s41598-025-01587-x. PMC12081920 40374761

[36] Ding S, Banerjee A, Burke SN, Hernandez AR. Time restricted feeding with or without ketosis influences metabolism-related gene expression in a tissue-specific manner in aged rats. Geroscience. 2025;47:4845–55. 10.1007/s11357-025-01632-7. PMC12181561 40153191

[37] Hatori M, Vollmers C, Zarrinpar A, DiTacchio L, Bushong EA, Gill S, et al. Time-restricted feeding without reducing caloric intake prevents metabolic diseases in mice fed a high-fat diet. Cell Metab. 2012;15:848–60. 10.1016/j.cmet.2012.04.019. PMC3491655 22608008

[38] Bray NL, Pimentel H, Melsted P, Pachter L. Near-optimal probabilistic RNA-seq quantification. Nat Biotechnol. 2016;34:525–7. 10.1038/nbt.3519. 27043002

[39] Soneson C, Love MI, Robinson MD. Differential analyses for RNA-seq: transcript-level estimates improve gene-level inferences. F1000Res. 2015;4:1521. 10.12688/f1000research.7563.2. PMC4712774 26925227

[40] Love MI, Huber W, Anders S. Moderated estimation of fold change and dispersion for RNA-seq data with DESeq2. Genome Biol. 2014;15:550. 10.1186/s13059-014-0550-8. PMC4302049 25516281

[41] Dyer SC, Austine-Orimoloye O, Azov AG, Barba M, Barnes I, Barrera-Enriquez VP, et al. Ensembl 2025. Nucleic Acids Res. 2025;53:D948–57. 10.1093/nar/gkae1071. PMC11701638 39656687

[42] Subramanian A, Tamayo P, Mootha VK, Mukherjee S, Ebert BL, Gillette MA, et al. Gene set enrichment analysis: a knowledge-based approach for interpreting genome-wide expression profiles. Proc Natl Acad Sci U S A. 2005;102:15545–50. 10.1073/pnas.0506580102. PMC1239896 16199517

[43] Bhuva D, Smyth G, Garnham A. msigdb: An ExperimentHub Package for the Molecular Signatures Database (MSigDB). R package version 1.14.0, https://davislaboratory.github.io/msigdb. 2024.

[44] Liberzon A, Birger C, Thorvaldsdottir H, Ghandi M, Mesirov JP, Tamayo P. The molecular signatures database (MSigDB) hallmark gene set collection. Cell Syst. 2015;1:417–25. 10.1016/j.cels.2015.12.004. PMC4707969 26771021

[45] Rath S, Sharma R, Gupta R, Ast T, Chan C, Durham TJ, et al. MitoCarta3.0: an updated mitochondrial proteome now with sub-organelle localization and pathway annotations. Nucleic Acids Res. 2021;49:D1541–7. 10.1093/nar/gkaa1011. PMC7778944 33174596

[46] Xu S, Hu E, Cai Y, Xie Z, Luo X, Zhan L, et al. Using clusterProfiler to characterize multiomics data. Nat Protoc. 2024;19:3292–320. 10.1038/s41596-024-01020-z. 39019974

[47] Badia IMP, Velez Santiago J, Braunger J, Geiss C, Dimitrov D, Muller-Dott S, Taus P, Dugourd A, Holland CH, Ramirez Flores RO, Saez-Rodriguez J. decoupleR: ensemble of computational methods to infer biological activities from omics data. Bioinform Adv. 2022;2:vbac016. 10.1093/bioadv/vbac016 PMC9710656 36699385

[48] Muller-Dott S, Tsirvouli E, Vazquez M, Ramirez Flores RO, Badia IMP, Fallegger R, et al. Expanding the coverage of regulons from high-confidence prior knowledge for accurate estimation of transcription factor activities. Nucleic Acids Res. 2023;51:10934–49. 10.1093/nar/gkad841. PMC10639077 37843125

[49] Wek RC, Anthony TG, Staschke KA. Surviving and adapting to stress: translational control and the integrated stress response. Antioxid Redox Signal. 2023;39:351–73. 10.1089/ars.2022.0123. PMC10443206 36943285

[50] Pakos-Zebrucka K, Koryga I, Mnich K, Ljujic M, Samali A, Gorman AM. The integrated stress response. EMBO Rep. 2016;17:1374–95. 10.15252/embr.201642195. PMC5048378 27629041

[51] Onat UI, Yildirim AD, Tufanli O, Cimen I, Kocaturk B, Veli Z, et al. Intercepting the lipid-induced integrated stress response reduces atherosclerosis. J Am Coll Cardiol. 2019;73:1149–69. 10.1016/j.jacc.2018.12.055. PMC6424590 30871699

[52] Kaspar S, Oertlin C, Szczepanowska K, Kukat A, Senft K, Lucas C, et al. Adaptation to mitochondrial stress requires CHOP-directed tuning of ISR. Sci Adv. 2021. 10.1126/sciadv.abf0971. PMC8153728 34039602

[53] Hoshino S, Kobayashi M, Higami Y. Mechanisms of the anti-aging and prolongevity effects of caloric restriction: evidence from studies of genetically modified animals. Aging (Albany NY). 2018;10:2243–51. 10.18632/aging.101557. PMC6188494 30222593

[54] Procaccini C, de Candia P, Russo C, De Rosa G, Lepore MT, Colamatteo A, et al. Caloric restriction for the immunometabolic control of human health. Cardiovasc Res. 2024;119:2787–800. 10.1093/cvr/cvad035. 36848376

[55] Czechowicz P, Wiech-Walow A, Koziol S, Dudzik J, Collawn JF, Bartoszewski R. A multilayered stress-response circuit: The mammalian mitochondrial UPR. FEBS J. 2026. 10.1111/febs.70607. PMC13580307 42216472

[56] Deota S, Lin T, Chaix A, Williams A, Le H, Calligaro H, et al. Diurnal transcriptome landscape of a multi-tissue response to time-restricted feeding in mammals. Cell Metab. 2023;35:150-165 e154. 10.1016/j.cmet.2022.12.006. PMC10026518 36599299

[57] Kang SG, Choi MJ, Jung SB, Chung HK, Chang JY, Kim JT, et al. Differential roles of GDF15 and FGF21 in systemic metabolic adaptation to the mitochondrial integrated stress response. iScience. 2021;24:102181. 10.1016/j.isci.2021.102181. PMC7920832 33718833

[58] Jena J, Garcia-Pena LM, Pereira RO. The roles of FGF21 and GDF15 in mediating the mitochondrial integrated stress response. Front Endocrinol (Lausanne). 2023;14:1264530. 10.3389/fendo.2023.1264530. PMC10561105 37818094

[59] Li T, Huang N, Chen H, Yang Y, Zhang J, Xu W, et al. Daytime-restricted feeding alleviates D-galactose-induced aging in mice and regulates the AMPK and mTORC1 activities. J Cell Physiol. 2025;240:e70020. 10.1002/jcp.70020. 40070151

[60] Di Pietrantonio N, Palmerini C, Pipino C, Baldassarre MPA, Bologna G, Mohn A, et al. Plasma from obese children increases monocyte-endothelial adhesion and affects intracellular insulin signaling in cultured endothelial cells: potential role of mTORC1-S6K1. Biochim Biophys Acta Mol Basis Dis. 2021;1867:166076. 10.1016/j.bbadis.2021.166076. 33422633

[61] Zhang N, Liu N, Zhao G, Yan J, Zhang P, Li X, et al. Effects of a two-week modified ketogenic diet on circulating lipoprotein subclasses, GDF15, and FGF21 in obese adults. J Transl Med. 2025;23:1244. 10.1186/s12967-025-07251-2. PMC12595818 41204286

[62] Plomgaard P, Hansen JS, Townsend LK, Gudiksen A, Secher NH, Clemmesen JO, et al. GDF15 is an exercise-induced hepatokine regulated by glucagon and insulin in humans. Front Endocrinol (Lausanne). 2022;13:1037948. 10.3389/fendo.2022.1037948. PMC9760804 36545337

[63] Chaiyasoot K, Khumkhana N, Deekum W, Chaichana C, Taweerutchana V, Srisuworanan N, et al. Alteration of BDNF, SPARC, FGF-21, and GDF-15 circulating levels after 1 year of anti-obesity treatments and their association with 1-year weight loss. Endocr Res. 2023;82:57–68. 10.1007/s12020-023-03435-2. PMC10462550 37436597

[64] Wang D, Townsend LK, DesOrmeaux GJ, Frangos SM, Batchuluun B, Dumont L, et al. GDF15 promotes weight loss by enhancing energy expenditure in muscle. Nature. 2023;619:143–50. 10.1038/s41586-023-06249-4. PMC10322716 37380764

[65] Richter MM, Thomsen MN, Skytte MJ, Kjeldsen SAS, Samkani A, Frystyk J, et al. Effect of a 6-week carbohydrate-reduced high-protein diet on levels of FGF21 and GDF15 in people with type 2 diabetes. J Endocr Soc. 2024;8:bvae008. 10.1210/jendso/bvae008. PMC10875725 38379856

[66] Song H, Yin D, Liu Z. GDF-15 promotes angiogenesis through modulating p53/HIF-1alpha signaling pathway in hypoxic human umbilical vein endothelial cells. Mol Biol Rep. 2012;39:4017–22. 10.1007/s11033-011-1182-7. 21773947

[67] Malik VA, Zajicek F, Mittmann LA, Klaus J, Unterseer S, Rajkumar S, et al. GDF15 promotes simultaneous astrocyte remodeling and tight junction strengthening at the blood-brain barrier. J Neurosci Res. 2020;98:1433–56. 10.1002/jnr.24611. 32170776

[68] Kim KH, Lee MS. GDF15 as a central mediator for integrated stress response and a promising therapeutic molecule for metabolic disorders and NASH. Biochimica et Biophysica Acta (BBA). 2021;1865:129834. 10.1016/j.bbagen.2020.129834. 33358864

[69] Asundi J, Zhang C, Donnelly-Roberts D, Solorio JZ, Challagundla M, Connelly C, et al. GDF15 is a dynamic biomarker of the integrated stress response in the central nervous system. CNS Neurosci Ther. 2024;30:e14600. 10.1111/cns.14600. PMC10867791 38357857

[70] Chen J, Kastroll J, Bello FM, Pangburn MM, Murali A, Smith PM, et al. Skeletal muscle mitochondrial dysfunction is associated with increased Gdf15 expression and circulating GDF15 levels in aged mice. Sci Rep. 2025;15:8101. 10.1038/s41598-025-92572-x. PMC11890589 40057594

[71] Breit SN, Brown DA, Tsai VW. The GDF15-GFRAL pathway in health and metabolic disease: friend or foe? Annu Rev Physiol. 2021;83:127–51. 10.1146/annurev-physiol-022020-045449. 33228454

[72] Wang D, Day EA, Townsend LK, Djordjevic D, Jorgensen SB, Steinberg GR. GDF15: emerging biology and therapeutic applications for obesity and cardiometabolic disease. Nat Rev Endocrinol. 2021;17:592–607. 10.1038/s41574-021-00529-7. 34381196

[73] Arumugam TV, Alli-Shaik A, Liehn EA, Selvaraji S, Poh L, Rajeev V, et al. Multiomics analyses reveal dynamic bioenergetic pathways and functional remodeling of the heart during intermittent fasting. Elife. 2023. 10.7554/eLife.89214. PMC10538958 37769126

[74] Williams AS, Crown SB, Lyons SP, Koves TR, Wilson RJ, Johnson JM, et al. Ketone flux through BDH1 supports metabolic remodeling of skeletal and cardiac muscles in response to intermittent time-restricted feeding. Cell Metab. 2024;36:422-437 e428. 10.1016/j.cmet.2024.01.007. PMC10961007 38325337

[75] Lanza IR, Zabielski P, Klaus KA, Morse DM, Heppelmann CJ, Bergen HR 3rd, et al. Chronic caloric restriction preserves mitochondrial function in senescence without increasing mitochondrial biogenesis. Cell Metab. 2012;16:777–88. 10.1016/j.cmet.2012.11.003. PMC3544078 23217257

[76] Perez-Rodriguez M, Huertas JR, Villalba JM, Casuso RA. Mitochondrial adaptations to calorie restriction and bariatric surgery in human skeletal muscle: a systematic review with meta-analysis. Metabolism. 2023;138:155336. 10.1016/j.metabol.2022.155336. 36302454

[77] Quiros PM, Mottis A, Auwerx J. Mitonuclear communication in homeostasis and stress. Nat Rev Mol Cell Biol. 2016;17:213–26. 10.1038/nrm.2016.23. 26956194

[78] Sparks LM, Redman LM, Conley KE, Harper ME, Yi F, Hodges A, et al. Effects of 12 months of caloric restriction on muscle mitochondrial function in healthy individuals. J Clin Endocrinol Metab. 2017;102:111–21. 10.1210/jc.2016-3211. PMC5413108 27778643

[79] Tang HW, Hu Y, Chen CL, Xia B, Zirin J, Yuan M, et al. The TORC1-regulated CPA complex rewires an RNA processing network to drive autophagy and metabolic reprogramming. Cell Metab. 2018;27(1040–1054):e1048. 10.1016/j.cmet.2018.02.023. PMC6100782 29606597

[80] Khan J, Pernicova I, Nisar K, Korbonits M. Mechanisms of ageing: growth hormone, dietary restriction, and metformin. Lancet Diabetes Endocrinol. 2023;11:261–81. 10.1016/S2213-8587(23)00001-3. 36848915

[81] Paoli A, Tinsley GM, Mattson MP, De Vivo I, Dhawan R, Moro T. Common and divergent molecular mechanisms of fasting and ketogenic diets. Trends Endocrinol Metab. 2024;35:125–41. 10.1016/j.tem.2023.10.001. 38577754

[82] Dey S, Baird TD, Zhou D, Palam LR, Spandau DF, Wek RC. Both transcriptional regulation and translational control of ATF4 are central to the integrated stress response. J Biol Chem. 2010;285:33165–74. 10.1074/jbc.M110.167213. PMC2963398 20732869

[83] Yuan JR, Tang J, Sheng R. The stress responsive transcription factor ATF4: from molecular structure to disease mechanisms. J Adv Res. 2026. 10.1016/j.jare.2026.03.017. 41831679

[84] Rodriguez-Colman MJ, Dansen TB, Burgering BMT. FOXO transcription factors as mediators of stress adaptation. Nat Rev Mol Cell Biol. 2024;25:46–64. 10.1038/s41580-023-00649-0. 37710009

[85] Gupta S, Afzal M, Agrawal N, Almalki WH, Rana M, Gangola S, et al. Harnessing the FOXO-SIRT1 axis: insights into cellular stress, metabolism, and aging. Biogerontology. 2025;26:65. 10.1007/s10522-025-10207-0. 40011269

[86] Klotz LO, Sanchez-Ramos C, Prieto-Arroyo I, Urbanek P, Steinbrenner H, Monsalve M. Redox regulation of FoxO transcription factors. Redox Biol. 2015;6:51–72. 10.1016/j.redox.2015.06.019. PMC4511623 26184557

[87] Eijkelenboom A, Burgering BM. FOXOs: signalling integrators for homeostasis maintenance. Nat Rev Mol Cell Biol. 2013;14:83–97. 10.1038/nrm3507. 23325358

[88] Hsieh PN, Fan L, Sweet DR, Jain MK. The Kruppel-like factors and control of energy homeostasis. Endocr Rev. 2019;40:137–52. 10.1210/er.2018-00151. PMC6334632 30307551

[89] Fan L, Sweet DR, Fan EK, Prosdocimo DA, Madera A, Jiang Z, et al. Transcription factors KLF15 and PPARdelta cooperatively orchestrate genome-wide regulation of lipid metabolism in skeletal muscle. J Biol Chem. 2022;298:101926. 10.1016/j.jbc.2022.101926. PMC9190004 35413288

[90] Yuce K, Ozkan AI. The kruppel-like factor (KLF) family, diseases, and physiological events. Gene. 2024;895:148027. 10.1016/j.gene.2023.148027. 38000704

[91] Ruberto AA, Grechez-Cassiau A, Guerin S, Martin L, Revel JS, Mehiri M, et al. KLF10 integrates circadian timing and sugar signaling to coordinate hepatic metabolism. Elife. 2021. 10.7554/eLife.65574. PMC8410083 34402428

[92] Eid W, Dauner K, Courtney KC, Gagnon A, Parks RJ, Sorisky A, et al. MTORC1 activates SREBP-2 by suppressing cholesterol trafficking to lysosomes in mammalian cells. Proc Natl Acad Sci U S A. 2017;114:7999–8004. 10.1073/pnas.1705304114. PMC5544314 28696297

[93] Lewis CA, Griffiths B, Santos CR, Pende M, Schulze A. Regulation of the SREBP transcription factors by mTORC1. Biochem Soc Trans. 2011;39:495–9. 10.1042/BST0390495. 21428927

[94] Peterson TR, Sengupta SS, Harris TE, Carmack AE, Kang SA, Balderas E, et al. MTOR complex 1 regulates lipin 1 localization to control the SREBP pathway. Cell. 2011;146:408–20. 10.1016/j.cell.2011.06.034. PMC3336367 21816276

[95] Duvel K, Yecies JL, Menon S, Raman P, Lipovsky AI, Souza AL, et al. Activation of a metabolic gene regulatory network downstream of mTOR complex 1. Mol Cell. 2010;39:171–83. 10.1016/j.molcel.2010.06.022. PMC2946786 20670887

[96] Laplante M, Sabatini DM. Regulation of mTORC1 and its impact on gene expression at a glance. J Cell Sci. 2013;126:1713–9. 10.1242/jcs.125773. PMC3678406 23641065

[97] Gulej R, Nagy D, Kristof R, Csiszar A, Patai R. Circulating factors as modifiable therapeutic targets in brain and cerebrovascular aging: insights from heterochronic parabiosis. Adv Transl Res. 2026. 10.1556/1661.2025.00107.

[98] Shimazu T, Hirschey MD, Newman J, He W, Shirakawa K, Le Moan N, et al. Suppression of oxidative stress by beta-hydroxybutyrate, an endogenous histone deacetylase inhibitor. Science. 2013;339:211–4. 10.1126/science.1227166. PMC3735349 23223453

[99] Chiang JYL, Ferrell JM. Bile acids as metabolic regulators and nutrient sensors. Annu Rev Nutr. 2019;39:175–200. 10.1146/annurev-nutr-082018-124344. PMC6996089 31018107

[100] Salminen A, Kaarniranta K, Kauppinen A. Integrated stress response stimulates FGF21 expression: systemic enhancer of longevity. Cell Signal. 2017;40:10–21. 10.1016/j.cellsig.2017.08.009. 28844867

[101] Ungvari Z, Tarantini S, Sorond F, Merkely B, Csiszar A. Mechanisms of vascular aging, a geroscience perspective: JACC focus seminar. J Am Coll Cardiol. 2020;75:931–41. 10.1016/j.jacc.2019.11.061. PMC8559983 32130929

[102] Ungvari Z, Tarantini S, Donato AJ, Galvan V, Csiszar A. Mechanisms of vascular aging. Circ Res. 2018;123:849–67. 10.1161/CIRCRESAHA.118.311378. PMC6248882 30355080

[103] Paneni F, Diaz Canestro C, Libby P, Luscher TF, Camici GG. The aging cardiovascular system: understanding it at the cellular and clinical levels. J Am Coll Cardiol. 2017;69:1952–67. 10.1016/j.jacc.2017.01.064. 28408026

[104] Ungvari Z, Tarantini S, Nyul-Toth A, Kiss T, Yabluchanskiy A, Csipo T, et al. Nrf2 dysfunction and impaired cellular resilience to oxidative stressors in the aged vasculature: from increased cellular senescence to the pathogenesis of age-related vascular diseases. Geroscience. 2019;41:727–38. 10.1007/s11357-019-00107-w. PMC6925097 31655958

[105] Fulop GA, Kiss T, Tarantini S, Balasubramanian P, Yabluchanskiy A, Farkas E, et al. Nrf2 deficiency in aged mice exacerbates cellular senescence promoting cerebrovascular inflammation. Geroscience. 2018;40:513–21. 10.1007/s11357-018-0047-6. PMC6294722 30470983

[106] Csiszar A, Gautam T, Sosnowska D, Tarantini S, Banki E, Tucsek Z, et al. Caloric restriction confers persistent anti-oxidative, pro-angiogenic, and anti-inflammatory effects and promotes anti-aging miRNA expression profile in cerebromicrovascular endothelial cells of aged rats. Am J Physiol Heart Circ Physiol. 2014;307:H292-306. 10.1152/ajpheart.00307.2014. PMC4121647 24906921

[107] Ungvari Z, Bailey-Downs L, Gautam T, Sosnowska D, Wang M, Monticone RE, Telljohann R, Pinto JT, De Cabo R, Sonntag WE, Lakatta EG. Age-associated vascular oxidative stress, Nrf2 dysfunction, and NF-κB activation in the nonhuman primate Macaca mulatta. Journals of Gerontology Series A: Biomedical Sciences and Medical Sciences. 2011Aug 1;66(8):866–75. 10.1093/gerona/glr092 PMC3148762 21622983

[108] Tarantini S, Valcarcel-Ares NM, Yabluchanskiy A, Fulop GA, Hertelendy P, Gautam T, et al. Treatment with the mitochondrial-targeted antioxidant peptide SS-31 rescues neurovascular coupling responses and cerebrovascular endothelial function and improves cognition in aged mice. Aging Cell. 2018. 10.1111/acel.12731. PMC5847870 29405550

